# Longitudinal assessment of chemotherapy-induced brain connectivity changes in cerebral white matter and its correlation with cognitive functioning using the GQI

**DOI:** 10.3389/fneur.2024.1332984

**Published:** 2024-02-07

**Authors:** Vincent Chin-Hung Chen, Wei Chuang, Yuan-Hsiung Tsai, Roger S. McIntyre, Jun-Cheng Weng

**Affiliations:** ^1^School of Medicine, Chang Gung University, Taoyuan, Taiwan; ^2^Department of Psychiatry, Chang Gung Memorial Hospital, Chiayi, Taiwan; ^3^Department of Medical Imaging and Radiological Sciences, and Department of Artificial Intelligence, Chang Gung University, Taoyuan, Taiwan; ^4^Department of Diagnostic Radiology, Chang Gung Memorial Hospital, Chiayi, Taiwan; ^5^Mood Disorder Psychopharmacology Unit, University Health Network, Department of Psychiatry, University of Toronto, Toronto, ON, Canada; ^6^Institute of Medical Science, University of Toronto, Toronto, ON, Canada; ^7^Departments of Psychiatry and Pharmacology, University of Toronto, Toronto, ON, Canada

**Keywords:** breast cancer, chemotherapy, generalized q-sampling imaging (GQI), cognitive function, longitudinal study

## Abstract

**Objective:**

Breast cancer was the most prevalent type of cancer and had the highest incidence rate among women worldwide. The wide use of adjuvant chemotherapy might have a detrimental effect on the human brain and result in chemotherapy-related cognitive impairment (CICI) among breast cancer patients. Furthermore, prior to chemotherapy, patients reported cancer-related cognitive impairment (CRCI), which might be due to physiological factors or mood symptoms. The present longitudinal study aimed to investigate microstructural and macroscale white matter alterations by generalized q-sampling imaging (GQI).

**Methods:**

The participants were categorized into a pre-chemotherapy group (BB) if they were diagnosed with primary breast cancer and an age-matched noncancer control group (HC). Some participants returned for follow-up assessment. In the present follow up study, 28 matched pairs of BB/BBF (follow up after chemotherapy) individuals and 28 matched pairs of HC/HCF (follow up) individuals were included. We then used GQI and graph theoretical analysis (GTA) to detect microstructural alterations in the whole brain. In addition, we evaluated the relationship between longitudinal changes in GQI indices and neuropsychological tests as well as psychiatric comorbidity.

**Findings:**

The results showed that disruption of white matter integrity occurred in the default mode network (DMN) of patients after chemotherapy, such as in the corpus callosum (CC) and middle frontal gyrus (MFG). Furthermore, weaker connections between brain regions and lower segregation ability were observed in the post-chemotherapy group. Significant correlations were observed between neuropsychological tests and white matter tracts of the CC, MFG, posterior limb of the internal capsule (PLIC) and superior longitudinal fasciculus (SLF).

**Conclusion:**

The results provided evidence of white matter alterations in breast cancer patients, and they may serve as potential imaging markers of cognitive changes. In the future, the study may be beneficial to create and evaluate strategies designed to maintain or improve cognitive function in breast cancer patients undergoing chemotherapy.

## Introduction

According to statistics published by the World Health Organization (WHO), breast cancer (BC) is the most prevalent type of cancer and had the highest incidence rate among women worldwide in 2020. Furthermore, the Taiwan Ministry of Health and Welfare reports that breast cancer has the highest incidence rate and is the third leading cause of cancer mortality in Taiwan (1). Breast cancer survivors generally complain of cognitive impairment, which has a negative impact on quality of life (2, 3).

There are several therapeutic regimens for breast cancer. These treatments include mastectomy, chemotherapy, hormone therapy, and radiotherapy. Among those therapies, chemotherapy-induced cognitive impairment (CICI) is one of the most well-known side effects in BC patients (2, 3). The wide use of adjuvant chemotherapy in breast cancer can induce emotional disturbance and cognitive impairment. Up to 75% of cancer survivors have experienced difficulties in multiple domains of cognition during treatment, and 35% of survivors experience these impairments for months or even years after treatment (4). The cause of CICI remains unclear; some articles attribute these symptoms to neurotoxicity (5–8). Moreover, a study investigated the concentration of a biomarker associated with axonal injuries in hematology, which was found to be 20 times higher in patients treated with chemotherapy than in controls (9).

Neuroimaging studies have shown white matter microstructural changes in BC survivors who are treated with chemotherapy. However, cognitive impairment affects 11%–35% of noncentral nervous system (non-CNS) cancer survivors prior to treatment (7, 10). Several neuroimaging studies have also revealed changes in multimodality brain abnormalities prior to treatment in BC patients, including gray matter volume, white matter volume, white matter microstructure, and structural as well as functional connectomic alterations. The mechanisms of this phenomenon remain unclear; some scholars ascribe these changes to increased cytokine levels in cancer patients, inflammation, or anesthesia during surgery (11–14). There is also an argument that breast cancer may act as a traumatic stressor leading to psychiatric comorbidity, which leads to cancer-related cognitive impairment (CRCI) (7, 15). To address the issue with non-CNS cancer patients, van der Willils et al. conducted a prospective study that conquers interference by psychological factors. After excluding negative feelings when diagnosed with cancer, they found no significant difference between patients and healthy controls (16). Deprez et al. revealed that there is no difference between breast cancer patients and noncancer controls after controlling for the factor of depression (17). Moreover, Menning et al. discovered lower white matter integrity and cognitive function in BC patients, which is related to fatigue (13). These findings emphasize the importance of negative feelings associated with the diagnosis that can influence brain structure.

The diffusion distribution varies along different tissue characteristics. White matter comprises axons that are sheathed by myelin, and the diffusion of water is restricted in white matter. Therefore, this structural magnetic resonance imaging (MRI) modality can detect white matter architecture and integrity based on diffusion anisotropy. Generalized q-sampling imaging (GQI) has been proposed for use in brain research (18). The basis of this model-free method is that the relationship between MR signals and underlying diffusion displacement is a Fourier transform (19). In 2010, GQI was introduced by Yeh, who derived a new relationship that can obtain the spin distribution function (SDF) from MR signals directly and is able to compare across voxels. This technique can solve the problem of crossing fibers and provide directional and quantitative information about fibers. In addition, unlike the QBI and DSI, which can only obtain datasets by shell sampling or grid sampling, respectively, the GQI is a general method. Both sampling schemes can be applied to GQI, which makes it more suitable for clinical use. Additionally, several indices are provided by the GQI, including generalized fractional anisotropy (GFA) and normalized quantitative anisotropy (NQA).

The human brain is such a complex structure that people devote time and effort to uncovering it. The human brain connectome is a comprehensive structural description of the brain network and provides insights into brain connections. One of the well-known methods of modeling the brain connectome is graph theory (20). People reconstruct brain graphs by defining specific regions of the brain as nodes (points) and structural or functional interconnections between regions as edges (lines). Subsequently, topological properties can be measured. Several studies have shown that combining the performance of topological properties obtained from brain graphs, the human brain is a small-world architecture. This means that the brain works with both segregated and integrated functions. Thus, we could evaluate the differences in brain networks on any topic (21, 22).

Previous noninvasive imaging studies have shown replicated results of functional and structural alterations in breast cancer patients. Based on our previous cross-sectional report (23), the aim of this longitudinal study was to use GQI, regarded as a technique that can handle complex fibers, to explore (1) longitudinal changes in white matter integrity and network connections in breast cancer survivors from pre- to postchemotherapeutic regimens and (2) the relationship between longitudinal changes in GQI indices and neuropsychological tests as well as psychiatric comorbidity. We hypothesized that mood symptoms and neurotoxicity would affect white matter tracts, especially in the dorsal attention network (DAN) and mode default mode network (DMN), which are involved in cognitive processing.

## Materials and methods

### Study design and participants

In the longitudinal study, all participants were recruited from Chiayi Chang Gung Memorial Hospital with approval by the Institutional Review Board (No. 104-5082B, 201700256B0, 201702027B0). All of them provided informed written consent, and all research was performed in accordance with relevant guidelines and regulations. Subjects were eligible if they met the following criteria: female, over the age of 20, and diagnosed with primary breast cancer. These patients were further categorized into pre-chemotherapy (BB) and post-chemotherapy groups (BBF). The most commonly used chemotherapeutic drugs in this study were epirubicin and docetaxel. If participants were pregnant, illiterate, had MRI contraindications, or were formerly diagnosed with major neurological illness, brain metastasis, they were excluded.

The study included 65 women with a history of BC who were scheduled to receive chemotherapy (BB) and 71 age-matched noncancer controls (HC). Some participants returned for follow-up assessment. Therefore, we had groups of BBF and HCF individuals. In the longitudinal study, 28 matched pairs of BB/BBF individuals and 28 matched pairs of HC/HCF individuals were included. At time point one (TP1), baseline data were collected after surgery but before the start of chemotherapy. At time point two (TP2), follow-up data took place at 7.5 months on average after completing chemotherapy. The assessment included completion of neuropsychological tests, psychiatric comorbidity, and diffusion MRI.

### Neuropsychological tests (NP tests)

Neuropsychological assessments were conducted by trained research assistants under the supervision of psychiatrists and clinical psychologists to obtain objective cognitive function in all participants.

1. Color Trails Test (CTT): The CTT, a language-free adaptation of the Trail Making Test, consists of circles numbered 1 to 25 in pink or yellow. CTT1 requires connecting circles sequentially, while CTT2 involves alternating between colors. CTT1 gauges sustained attention, and CTT2 evaluates cognitive flexibility. Scores are based on completion times in seconds (24, 25).
2. Digit Symbol Substitution (DSS): Processing speed is assessed with the DSS subset of the Wechsler Adult Intelligence Scale-III (WAIS-III). Test-takers match symbols to digits within a set time of 120 s, with scores reflecting the number of correct pairs (26).

### Patient-reported cognitive function and mood symptoms (PR)

To comprehend the subjective measures of all subjects, they were also asked to fill out several questionnaires to assess their subjective cognitive function and mood symptoms, including the FACT-Cog, PHQ and HADS-A.

1. Functional assessment of cancer therapy cognitive version 3 (FACT-cog): FACT-cog is used to measure multiple cognitive domains self-reports and impact on quality of life in the cancer population. The questionnaire consists of 37 questions on a 5-point Likert scale (0–4) belonging to 4 subscales: perceived cognitive impairment (CogPCI, score range: 0–80), perceived cognitive ability (CogPCA, score range: 0–36), cognitive impairment by others (CogOth, score range: 0–16) and impact on quality of life (CogQoL, score range: 0–16). Lower scores on this scale indicate more severe cognitive impairment (27).
2. Patient Health Questionnaire-9 (PHQ-9): The PHQ-9, designed to identify depressive disorders, assesses nine symptoms from the Diagnostic and Statistical Manual of Mental Disorders (DSM-IV) criteria on a 4-point Likert scale (0–3) per item. Scores of 5, 10, 15, and 20 indicate mild, moderate, moderately severe, and severe depression, respectively (28).
3. Anxiety subscale of the Hospital Anxiety and Depression Scale (HADS-A): The 14-item HADS is a self-report questionnaire consisting of two subscales: depression and anxiety. Each subscale contains 7 items on a 4-point Likert scale (0–3). The authors suggest that in each subscale, a score of 0 to 7 could be regarded as normal, a score of 8 to 10 being doubtful case and a score more than 11 indicates mood disorder (29). In this study, we evaluated anxiety by using the anxiety subscale of the HADS.

### Diffusion imaging acquisition

Diffusion MRI data were acquired by using 3 T MRI (Verio, Siemens) at Chiayi Chang Gung Memorial Hospital. A single-shot spin echo echo-planar imaging (SE-EPI) pulse sequence was performed to obtain diffusion-weighted images in 193 diffusion-sensitive gradients with b-values of 0, 1,000, 1,500, and 2,000 s/mm^2^ along the 1, 64, 64, and 64 directions, respectively. Constant parameters were repetition time (TR)/echo time (TE) = 8943/115 ms, field of view (FOV) = 250 × 250 mm^2^, slice thickness = 4 mm, matrix size = 128 × 128, voxel size = 1.9 × 1.9 × 3.4 mm^3^, number of excitations (NEX) = 1, and number of slices = 35. It took approximately 30 min to complete the examination.

### Imaging processing

Diffusion-weighted imaging (DWI) is vulnerable to several artifacts, such as susceptibility, eddy current distortion, and motion. Consequently, the first step of our study is to perform eddy current correction by using the diffusion toolbox of the FMRIB software library (FSL, Analysis Group, Oxford, United Kingdom) to ensure image quality (30). After that, Statistical Parametric Mapping (SPM12, The Wellcome Centre for Human Neuroimaging, London) was applied for normalization and affine nonlinear registration into Montreal Neurological Institute (MNI) space (31). Finally, normalized DWI data were reconstructed with the GQI method. The GQI indices, including GFA maps and NQA maps, and connectivity matrices were obtained from DSI studio (NTU, Taipei, Taiwan) for further analysis (18).

### Statistical analysis

Two kinds of statistical approaches, voxel-based analysis (VBA) and graph theoretical analysis (GTA), were both performed in research designs. Demographic data, NP tests, and PR were analyzed in SPSS25. The critical alpha value was set as 0.05 corrected by the false discovery rate (FDR) for the significance level of all statistical analyses.

### Longitudinal study

A voxel-based two-way repeated-measures ANCOVA model was applied to GFA and NQA whole-brain maps, with time as the within-subject factor and group as the between-group factor, to investigate group-by-time interactions. Paired t tests were used to assess the statistically significant changes in the GQI indices from TP1 (BB and HC) to TP2 (BBF and HCF). In addition, a general linear model was performed to explore the relationship between neuropsychological tests/PRO difference scores (BBF-BB), including ΔCTT, ΔDSS, ΔFACT-Cog, ΔPHQ9, and ΔHADS-A, and changes in GQI index values (BBF-BB), including ΔGFA and ΔNQA. Age and years of education were treated as covariates of no interest to avoid individual differences. In this study, we adopted not only multiple domains of objective cognition, including attention, executive function, and processing speed but also subjective self-reported cognitive function and mood symptoms. Values exceeding 3 times interquartile ranges (extreme outliers) were excluded from the study. These procedures were completed in SPM. Furthermore, the significant correlations of multiple regression were presented with scatter plots by extracting values of the specific voxel in all subjects.

For network measurement, we used connectomics based on whole-brain tractography to obtain connectivity matrices. Fiber tracking was terminated if NQA < 0.15 or angular threshold>70 degrees. The brain parcellation scheme was based on the automated anatomical labeling (AAL) atlas, and the cerebral cortex was divided into 90 nodes. Since the quantification of the structural connectome is problematic, we made some corrections (32). The counts of fiber track interconnecting nodes were unreliable due to the homogeneous seeding strategy. That is, longer fibers with more seeds will reconstruct more streamlines. Consequently, the inverse length of the fiber tracks was applied to scale the overestimated counts. To clarify whether the counts indicated biological measures, we weighted the normalized counts with NQA to emphasize the strength of the connections. In brief, the edges were defined as counts of fiber tracks divided by length and multiplied by NQA. Eventually, we obtained a 90×90 connectivity matrix from each participant. Subsequently, these connectivity matrices were analyzed by using Graph Analysis Toolbox (GAT, Stanford University, Stanford, CA, United States) and Network-based statistics (NBS, University of Melbourne, Australia) (33, 34).

Topological properties were acquired from GAT, and the 10 parameters listed below were chosen for subsequent analysis: assortativity, modularity, transitivity, global efficiency, mean local efficiency, characteristic path length, model-based clustering coefficient, lambda (normalized shortest path length), gamma (normalized clustering coefficient), and sigma (small-worldness). The area under the curve (AUC) of each parameter within the selected density range (0.06–0.22, density interval = 0.01) was calculated to compare between groups. The density represents the fraction of current counts of edges out of all possible connections.

To identify structural subnetwork differences between groups, a nonparametric approach of 5,000 permutations testing with the general linear model (GLM) was performed in NBS. Then, the significant results reported by NBS were visualized with the BrainNet Viewer (National Key Laboratory of Cognitive Neuroscience and Learning, Beijing Normal University, Beijing, China) (35).

## Results

### Demographic characteristics of the longitudinal study population

In the longitudinal study, only matched participants between TP1 and TP2 were included. A total of 56 pairs of participants were recruited from Chiayi Chang Gung Memorial Hospital. Baseline assessment was conducted after surgery and before the start of chemotherapy. The follow-up assessment occurred 7.5 months after the last cycle of chemotherapy. From baseline to follow-up assessment. Anxiety significantly decreased in the patient group, which was not observed in the control group. The average scan time intervals were 406 and 552 days in the patient group and control group, respectively. An overview of demographic characteristics is provided in Table 1. Moreover, the mood symptoms and cognitive performance in the patient group are reported in Table 2. Remarkably lower scores were observed in the HADS-A, FACT-cog, and CogPCI in the patient group after receiving chemotherapy.

**Table 1:** Demographic characteristics of longitudinal design.

	BB (*n* = 28)	BBF (*n* = 28)	HC (*n* = 28)	HCF (*n* = 28)	Mann–Whitney test
	Mean/No. (SD)	Mean/No. (SD)	Mean/No. (SD)	Mean/No. (SD)	*p*
Age (years)	52.29 (8.11)	52.75 (8.29)	48.61 (8.88)	49.46 (8.6)	*–*
Education (years)	11.61 (4.51)	11.71 (4.48)	12.48 (4.28)	12.55 (4.20)	–
Days after last cycle of chemotherapy (days)	–	226 (251)	–	–	–
TP1 ~ TP2 interval (days)	406 (273)		552 (189)		<0.001*

*p < 0.05. Please look for the abbreviation details in the Appendix.

**Table 2:** NP tests and PR of the patient group.

	BB (*n* = 28)	BBF (*n* = 28)	Wilcoxon signed ranks test	Effect size
	Mean/No. (SD)	Mean/No. (SD)	*p*	*d*
PHQ9	3.68 (2.72)	2.82 (2.36)	0.06	0.338
HADS-A	3.21 (2.91)	1.35 (1.87)	<0.001*	0.760
CTT1	49.51 (16.18)	50.18 (25.77)	0.412	0.031
CTT2	95.22 (30.82)	99.41 (46.95)	0.802	0.106
DSS	63.54 (18.48)	64.11 (19.19)	0.799	0.030
FACT_cog	120.71 (11.45)	115.39 (11.88)	0.015*	0.456
CogPCI	67.14 (6.69)	63.61 (7.20)	0.016*	0.508
CogPCA	23.04 (4.42)	21.29 (3.61)	0.064	0.434
CogOth	15.54 (1.04)	15.61 (0.83)	0.668	0.074
CogQoL	15.00 (2.28)	14.89 (2.55)	0.554	0.045

*p < 0.05. Please look for the abbreviation details in the Appendix.

### Voxel-based analysis (VBA)

**Figure 1:**
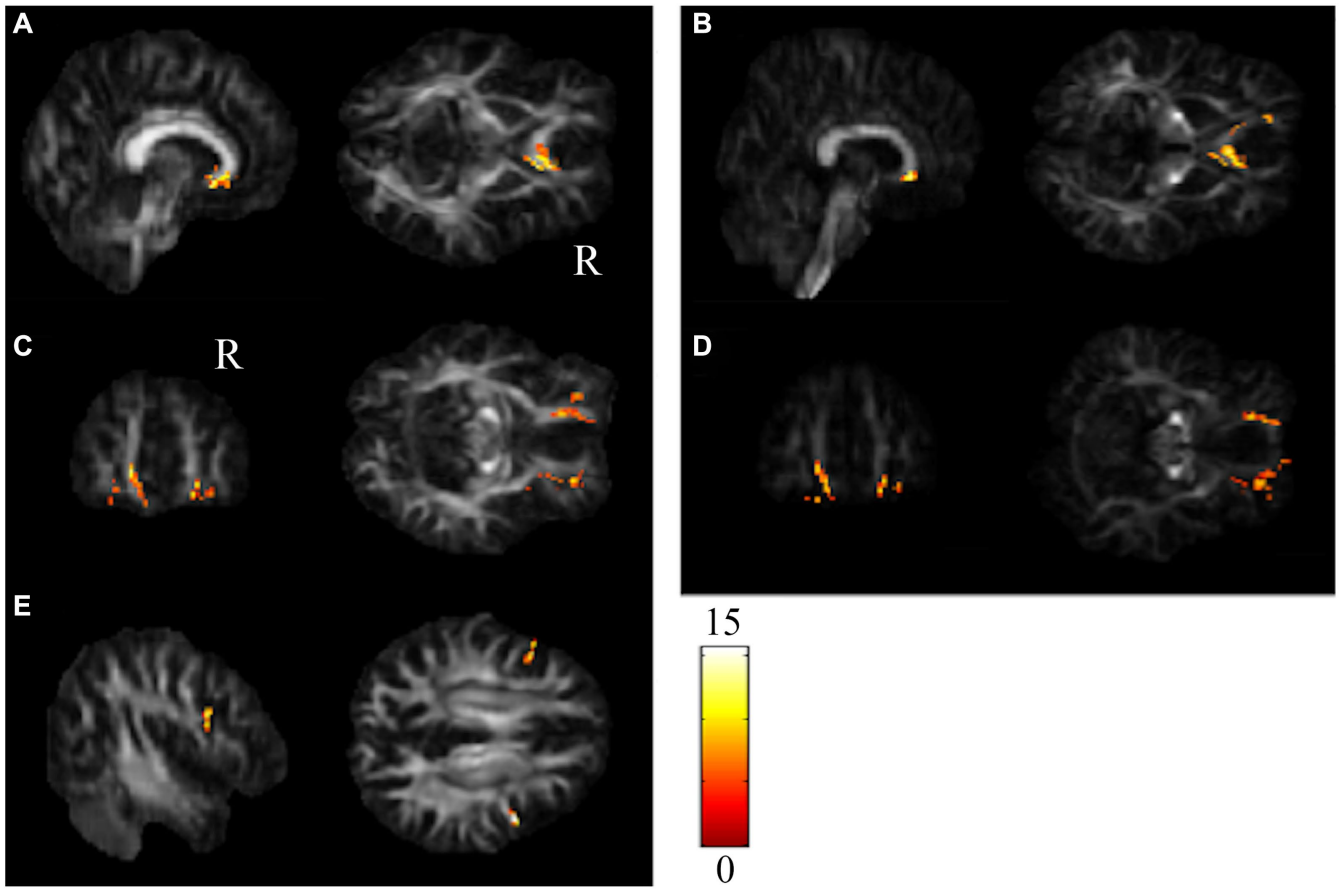
Group by time interaction assessed by repeated-measures ANCOVA. Significant group-by-time interactions were observed in **(A,B)** CC, **(C,D)** bi-ORBmidF, and **(E)** bi-MFG. The left column shows the results of GFA, and the right column shows the results of NQA (α = 0.05). The color bar represents F-scores. Please see the abbreviation details in the Appendix.

**Figure 2:**
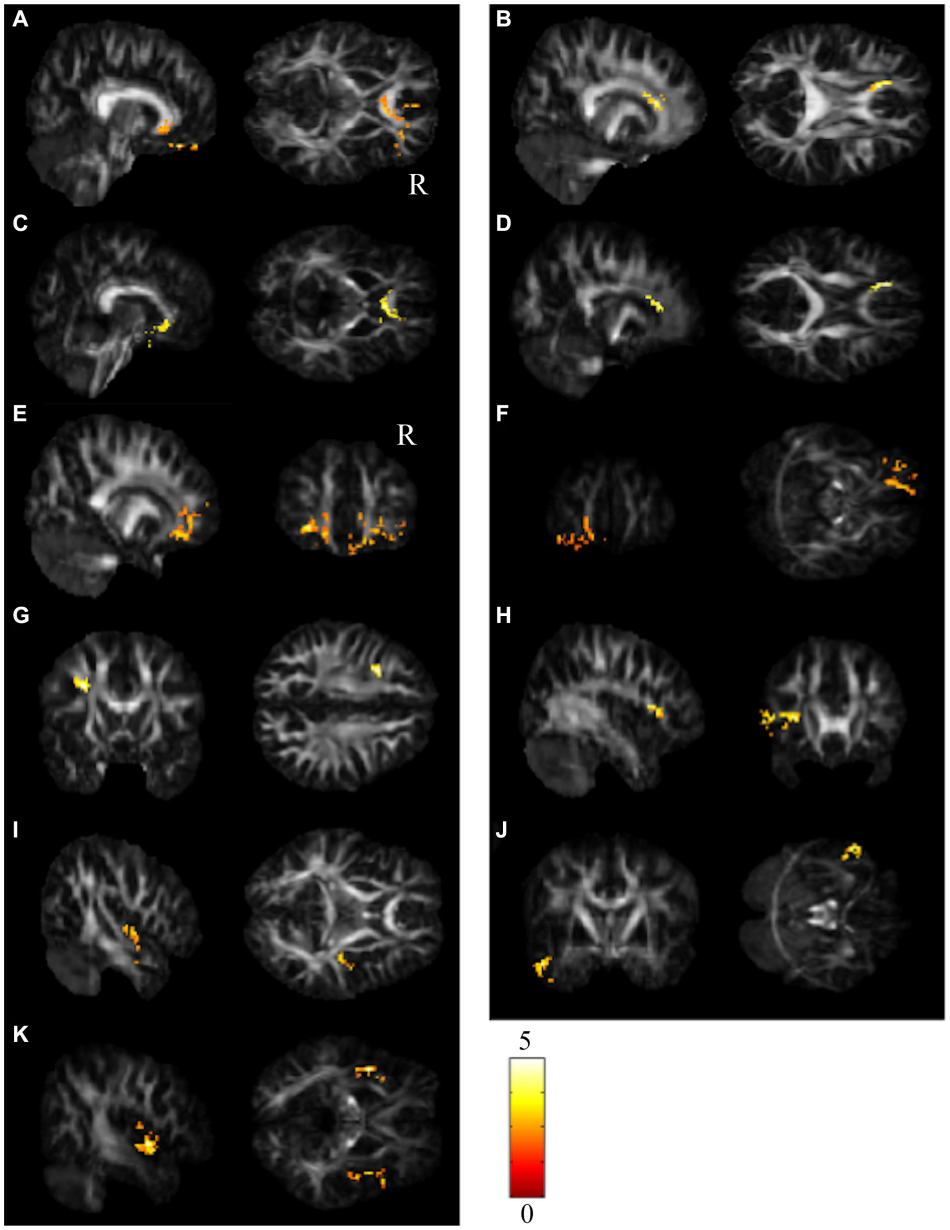
Results of paired t test between the BB and BBF groups. After receiving chemotherapy, patients showed GQI index reductions in the **(A–D)** CC, **(E,F)** l-ORBmidF, **(G)** l-MFG, **(H)** l-IFG, **(I)** r-STG, **(J)** l-MTG and **(K)** bi-insula. The results of differences in GFA are presented in **(A,B,E,G–I)**. The others differed in NQA (α = 0.05). The color bar represents *t*-scores of *t*-statistics. Please see the abbreviation details in the Appendix.

**Figure 3:**
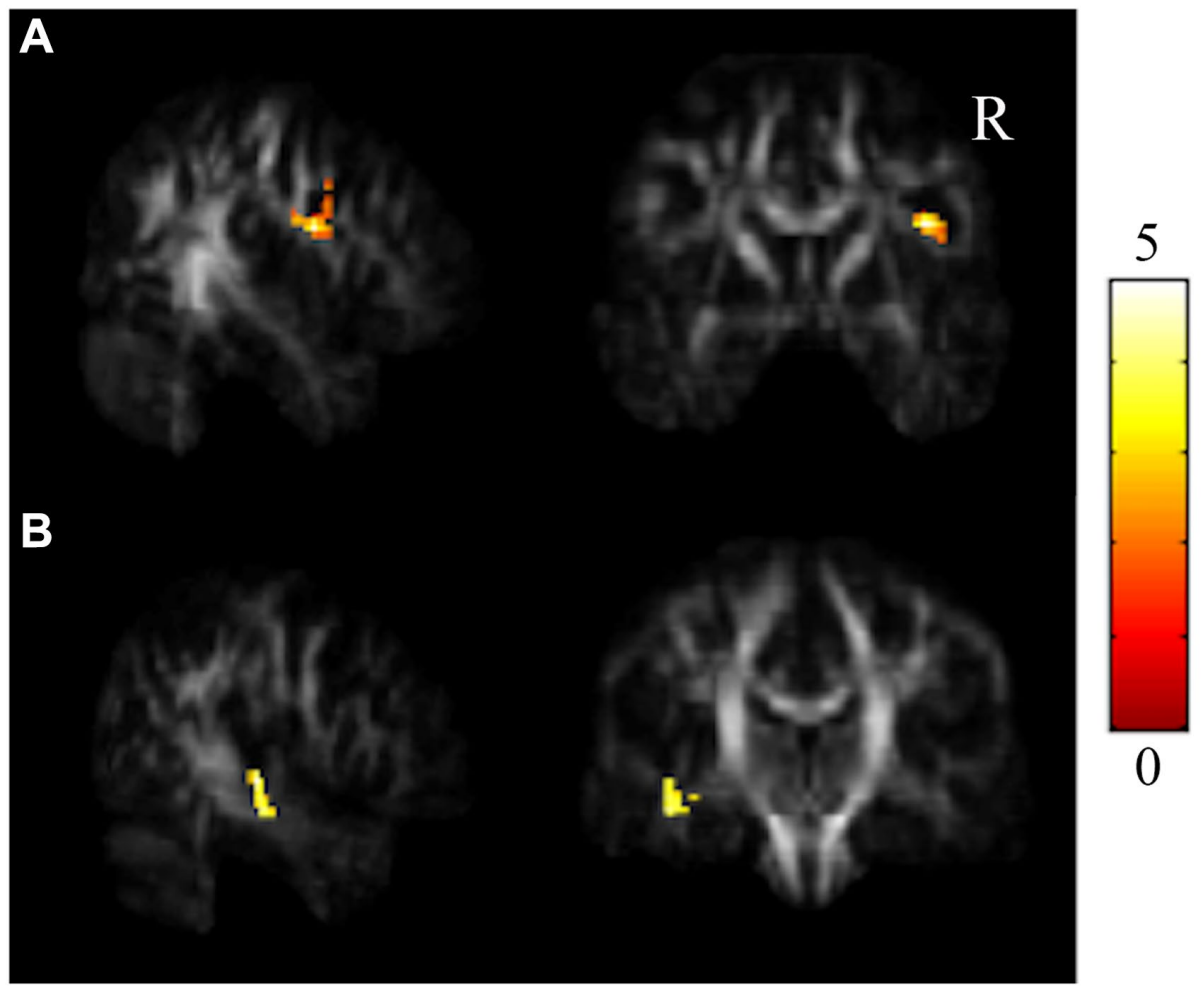
Results of paired *t* tests between the HC and HCF groups. There were significant decreases in NQA in **(A)** r-IFG and **(B)** l-STG in the control group from TP1 to TP2 (α = 0.05). The color bar represents *t*-scores of *t*-statistics. Please see the abbreviation details in the Appendix.

The repeated-measures ANCOVA explored significant group by time interactions in the regions of the corpus callosum (CC), bilateral orbital part of the middle frontal gyrus (ORBmidF), and bilateral middle frontal gyrus (MFG) (Figure 1, Supplementary Table S1). Paired t tests detected significantly decreased GQI indices in the regions of the CC, left ORBmidF, MFG, left inferior frontal gyrus (IFG), right superior temporal gyrus (STG), left middle temporal gyrus (MTG) and bilateral insula in the patient group (Figure 2, Supplementary Table S1) and notably lower GFA values in the regions of the right IFG and left STG in the control group (Figure 3, Supplementary Table S1) from TP1 to TP2.

### Graph theoretical analysis (GTA)

**Figure 4:**
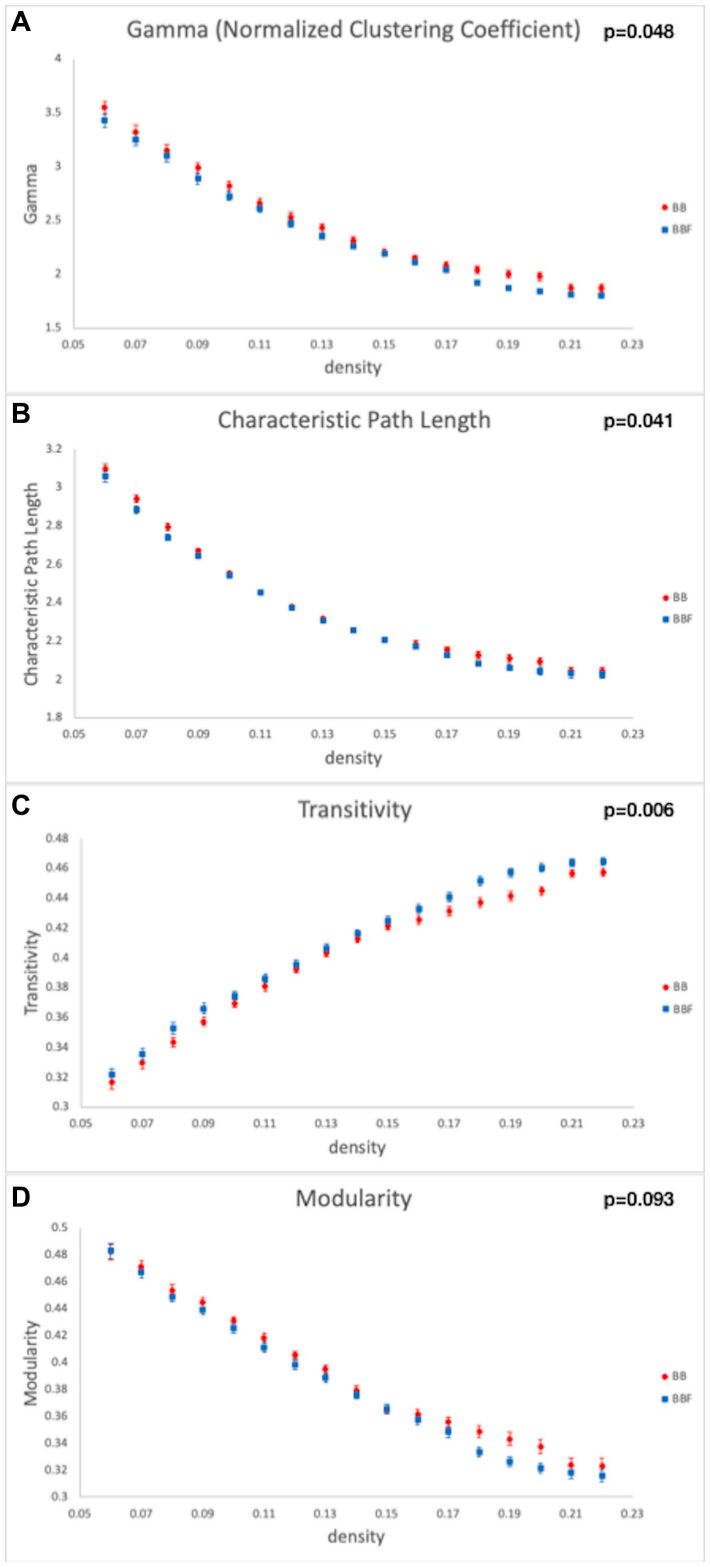
Topological data analysis results in the longitudinal study design. Topological properties differed in **(A)** gamma, **(B)** characteristic path length, **(C)** transitivity, and **(D)** modularity between the BB and BBF groups.

**Figure 5:**
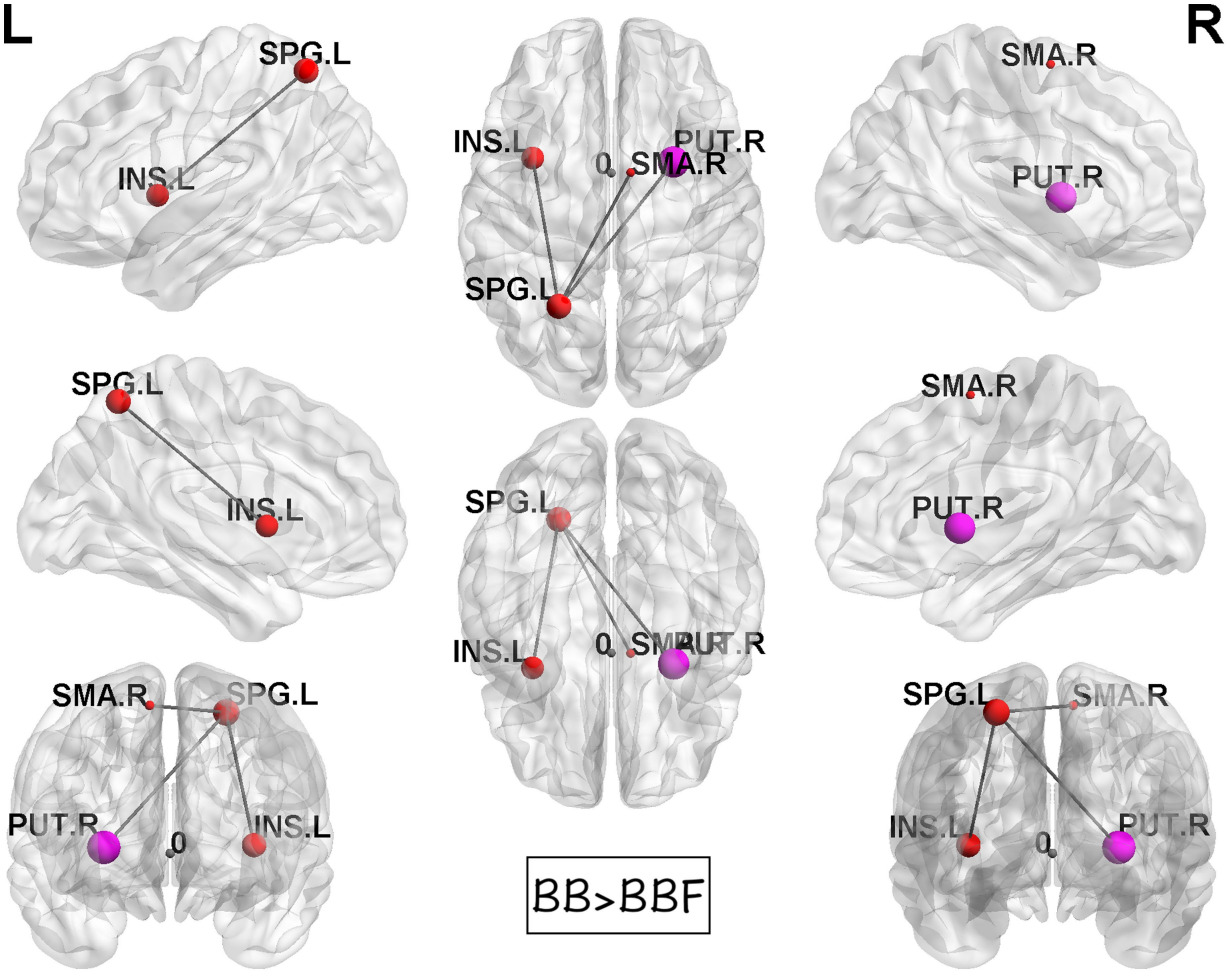
Results of NBS between the BB and BBF groups. There were significantly weaker connections among the supplementary motor area, parietal lobe, and subcortical regions in the patient group from TP1 to TP2.

While computing the topological properties, we integrated the AUC of each parameter by extracting the density range from 0.06 to 0.22. The topological data analysis revealed that the AUC differed significantly in some topological properties between BB and BBF but not between HC and HCF. The results showed significantly lower gamma, lower characteristic path length, higher transitivity, and nearly significantly lower modularity in the post-chemotherapy group than in the pre-chemotherapy group (Figure 4). Additionally, NBS detected significantly weaker structural connections among the supplementary motor area, parietal lobe and subcortical regions in chemotherapy-treated patients than at baseline (*p* = 0.025; Figure 5).

### Correlation analysis

**Figure 6:**
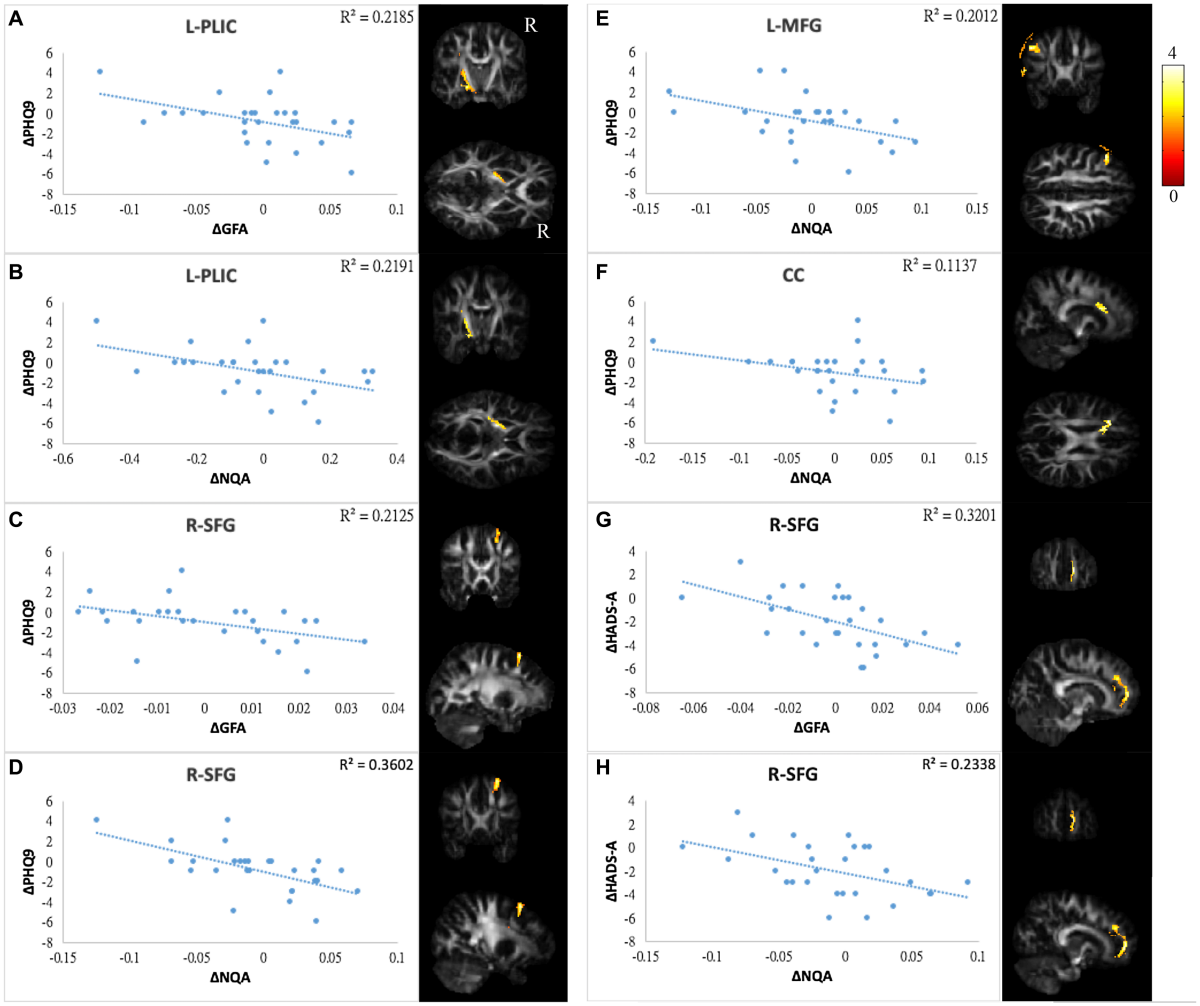
Correlations between GQI indices and mood symptoms. Scatter plot and *t*-score of *t*-statistics overlapping on GFA/NQA map illustrated the negative correlations between changes in PHQ9 and changes in **(A,B)** GFA/NQA of l-PLIC, **(C,D)** GFA/NQA of r-SFG, **(E)** NQA of l-MFG, and **(F)** NQA of CC. In addition, negative correlations were found between HADS-A and **(G)** GFA as well as **(H)** NQA of r-SFG. The color bar represents *t*-scores. Please see the abbreviation details in the Appendix.

**Figure 7:**
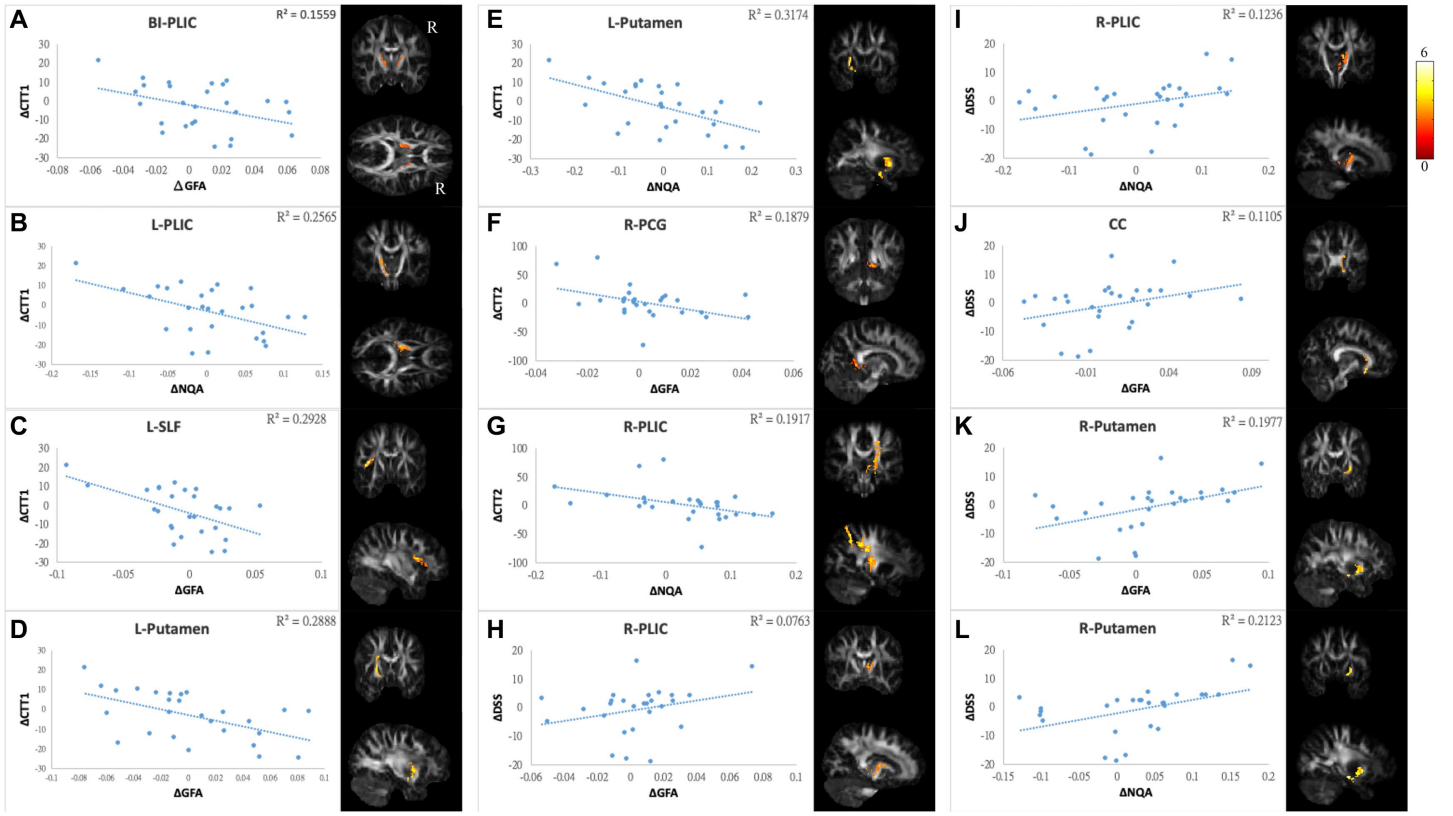
Correlations between GQI indices and objective cognitive function. The results of NP tests showed a negative correlation between differences in **(A)** CTT1 and GFA of bi-PLIC, **(B)** CTT1 and NQA of l-PLIC, **(C)** CTT1 and GFA of l-SLF, **(D,E)** CTT1 and GFA/NQA of l-putamen, **(F)** CTT2 and GFA of r-PCG, and **(G)** CTT2 and NQA of r-PLIC. Additionally, there were positive correlations between alterations in DSS and **(H,I)** GFA/NQA of the r-PLIC, **(J)** GFA of the CC, and **(K,L)** GFA/NQA of the r-putamen. The color bar represents *t*-scores. Please see the abbreviation details in the Appendix.

**Figure 8:**
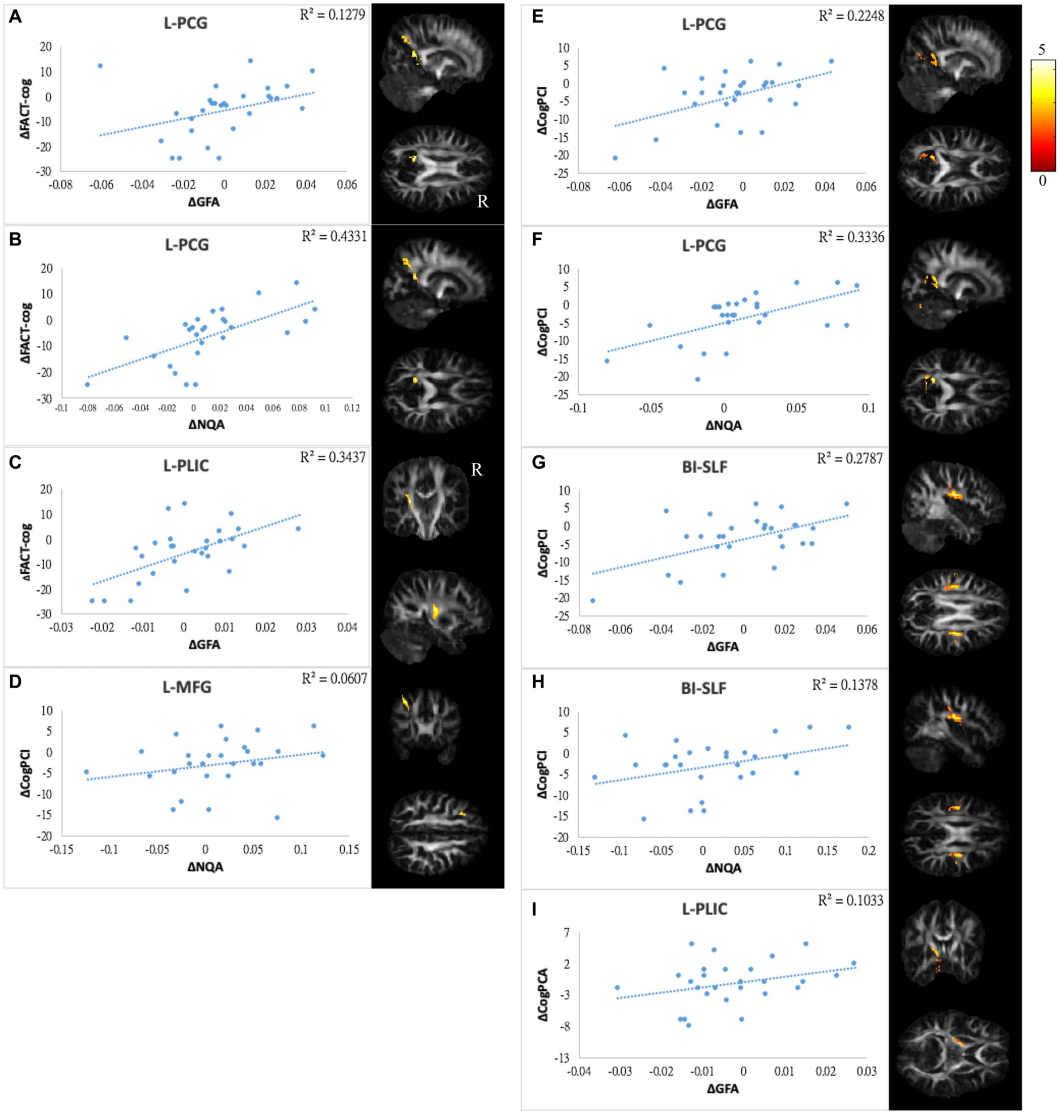
Correlations between GQI indices and subjective cognitive function. The results of subjective cognitive performance showed positive correlations between changes in **(A,B)** FACT-cog and GFA/NQA of l-PCG, **(C)** FACT-cog and GFA of l-PLIC, **(D)** CogPCI and NQA of l-MFG, **(E,F)** CogPCI and GFA/NQA of l-PCG, **(G,H)** CogPCI and GFA/NQA of bi-SLF, and **(I)** CogPCA and l-PLIC. The color bar represents *t*-scores. Please see the abbreviation details in the Appendix.

The differences in GQI indices and changes in score in NP tests/PR from TP1 to TP2 were used for correlation tests. Values exceeding 3 times interquartile ranges (extreme outliers) were excluded from the present study. The correlation analysis was not performed on CogOth and CogQoL due to nearly no changes in each patient at two time points. Finally, there were 27 to 28 pairs of patients included in each assessment. A summary of the correlation results is provided in Supplementary Table S2, and the scatter plots of these correlations are illustrated in Figures 6–8.

In mood symptoms, the present study detected that changes in PHQ9 were negatively correlated with changes in GQI indices in the left posterior limb of the internal capsule (PLIC), right superior frontal gyrus (SFG), left MFG, and CC (Figures 6A–F). The differences in HADS-A were negatively correlated with alterations in GQI indices in the right SFG as well (Figures 6G,H). For objective cognitive function, increasing the duration to complete CTT1 was associated with a reduction in GQI indices in the bilateral PLIC, left superior longitudinal fasciculus (SLF), and left putamen (Figures 7A–E). The phenomenon appeared in CTT2 in the right posterior cingulate gyrus (PCG) and right PLIC as well (Figures 7F,G). Positive correlations were found between changes in DSS and changes in GQI indices in the right PLIC, CC, and right putamen (Figures 7H–L). For subjective cognitive function, reducing the sum of the FACT-cog scores was positively related to decreased GQI indices in the left PCG and left PLIC (Figures 8A–C). There were positive correlations between changes in the CogPCI subscale and changes in GQI indices in the left MFG, left PCG and bilateral SLF (Figures 8D–H). In addition, diminishing in the CogPCA subscale was positively related to declining GQI indices in the left PLIC (Figure 8I).

## Discussion

In this study, we found several white matter tract disruptions associated with the DMN in breast cancer patients after receiving chemotherapy. Furthermore, the longitudinal changes from pre- to post-chemotherapy in white matter were related to alterations in mood symptoms and cognition. Previous studies have reported that whether breast cancer patients received chemotherapy or not, they suffered from cognitive function impairment. Cancer, stress, and various therapeutic strategies might have an impact on the brain. These adverse effects could affect memory, attention, executive function, and processing speed (3, 6, 7).

In a recent study employing MRI voxel-based morphometry (VBM), Chen et al. detected lower gray matter density, and Koppelmans et al. showed smaller gray matter as well as total brain volume in breast cancer patients after completion of chemotherapy (36, 37). Other research discovered a decline in regional gray matter density, encompassing the bilateral frontal, temporal, and cerebellar regions, as well as the right thalamus, among breast cancer patients from baseline to one month after undergoing chemotherapy. Nevertheless, some regions exhibited signs of recovery at the one-year mark post-chemotherapy (38).

In addition to VBM, other imaging modalities have reported structural and functional brain alterations in patients treated with chemotherapy. The researchers have examined CICI by using diffusion tensor imaging (DTI). The first DTI-based study of CICI in breast cancer patients was published by Abraham et al., who reported white matter integrity reduction in the genu of the CC (39). Subsequent studies report widespread disruption in white matter microstructure, and these injuries are correlated with cognitive impairments. Furthermore, there is a greater severity of affectation in the CC, SLF, and frontal areas. It appears that these cognitive impairments originate from a subtle but diffuse brain injury, as shown in neuroimaging studies (11, 40–43). Another investigation conducted by Deprez et al. utilizing DTI analysis identified a significant reduction in brain fractional anisotropy (FA) in the frontal, parietal, and occipital white matter tracts in breast cancer patients 3 to 4 months post-chemotherapy, correlating with changes in attention and verbal memory performance compared to baseline (17). Kaiser et al. and Pomykala et al. concluded from their studies that structural brain imaging, incorporating volumetric and DTI analyses, disclosed diminished gray and white matter volume, as well as reduced white matter integrity persisting from months to years after chemotherapy. This supports the proposition that structural brain alterations observed post-chemotherapy are indeed a consequence of exposure to chemotherapeutic agents (44, 45). Another investigation, comparing the brains of breast cancer survivors exposed to chemotherapy with those of healthy women, assessed the long-term impact of chemotherapy on brain microstructural integrity. Utilizing tract-based spatial statistics for DTI analysis, the authors employed linear regression to explore the influence of the time elapsed since chemotherapy. The findings revealed an inverse association between the length of time post-chemotherapy and FA, mean diffusivity (MD), and radial diffusivity (RD) among breast cancer survivors. The authors reported an adverse effect of adjuvant chemotherapy on the white matter microstructure integrity in breast cancer survivors who had survived for an average of more than 20 years (46).

In addition to structural methodologies, a majority of functional MRI (fMRI) studies focusing on tasks related to breast cancer (such as visual memory, verbal memory, attention, and executive functioning tasks) have identified alterations in multifocal cerebral cortical activities and functional networks among breast cancer survivors. These alterations varied depending on the specific task designs (47–51). McDonald et al., utilizing task-based fMRI, observed reduced activation related to working memory in the frontal lobe among breast cancer patients one-month post-chemotherapy, with partial recovery noted at the one-year mark (48). Furthermore, Dumas et al. reported decreased functional connectivity in the dorsal attention network among breast cancer patients one month after chemotherapy, partially returning to baseline at one year. However, reduced connectivity was observed in the default mode network at both one month and one year following chemotherapy (51). Using resting-state fMRI (rs-fMRI) and seed-based correlation analysis (SCA), Miao et al. discovered diminished functional connectivity in the dorsal medial prefrontal cortex and medial temporal lobe subsystems, potentially linked to the attention function of breast cancer patients one month after chemotherapy (52). Similarly, Wang et al. found decreased functional connectivity in the dorsolateral prefrontal cortex and inferior frontal gyrus, correlating with executive deficits in breast cancer patients one-month post-chemotherapy (53). Notably, Miao et al. also identified decreased functional connectivity in the anterior cingulate cortex among breast cancer patients three years after chemotherapy, correlating with impaired executive function (54). Moreover, functional connectivity was observed to decrease in the DMN (50). Nevertheless, white matter recovery may occur after a period of completion of chemotherapy. Deprez et al. reported white matter integrity reduction after 3–5 months of completion of chemotherapy, whereas longitudinal recovery was observed after 3–4 years of completion of chemotherapy.

### Voxel-based analysis

In the study, both microstructure and macroscale white matter alterations in breast cancer patients were found after completion of chemotherapy. In longitudinal studies, individual biases were excluded. We observed that the GQI indices decreased in the CC, middle and inferior frontal gyrus, superior and middle temporal gyrus, and insula in the post-chemotherapy assessment. However, reductions in the IFG and STG were also found in the control group. We could not differentiate whether aging or therapeutic regimens contributed to the results (55, 56).

Except for the IFG and STG, the observations in the CC and MFG were consistent with the cross-sectional design (57). We further found a significant decrease in the MTG. Neurotoxicity has been shown to have a detrimental effect on the temporal gyrus, and it is associated with working memory and recognition. Several studies have also detected abnormalities in the MTG (36, 40, 48, 57). In addition, we revealed a rarely reported region of the insula in the post-chemotherapy group. The insula is connected to other brain regions through the corona radiata, CC, superior and inferior longitudinal fasciculus, and external capsule (58). This structure is believed to play a role in controlling cognitive functions, including emotion, sensorimotor processing, and self-awareness, which are related to psychopathology. Furthermore, it can integrate internal and external information to coordinate the switch between the DMN and central executive network (CEN) (59). A longitudinal study showed that gray matter density was reduced in the insula in older women with breast cancer (>60 years old). They concluded that older women might be more vulnerable to chemotherapy (36). The mean age of our study was approximately 52.5 years old, falling between those of former studies. Moreover, brain activity has been identified to be altered in breast cancer patients with chemotherapy in our previous fMRI data (60). The insula is a structure often mentioned as related to anxiety. However, the patients are less anxious after receiving chemotherapy. Therefore, this finding could not be explained by anxiety and might result from chemotherapy. We believe that this result was not a coincidence, and it is worthwhile to investigate these findings in future work.

In line with our microstructure findings, we illustrated weaker subnetwork interconnections in the post-chemotherapy group. The involved regions of the hippocampus, precuneus, insula, frontal, parietal, and temporal lobes were sections of the DMN. Our previous fMRI study discovered similar functional connectivity changes. Feng et al. explored hippocampal connectivity alterations induced by chemotherapy in breast cancer survivors as well (61).

### Network-based statistical analysis

Voxel-based and network-based statistics identified that white matter integrity and connections decreased after chemotherapy. Whole-brain topological analysis suggested that they remained a small-world network. However, there were still some differences in the efficient network. There was a significant increase in transitivity, a decrease in the normalized clustering coefficient, and a marginally significant decrease in modularity. These three indices are typically categorized in terms of segregation. A higher value indicates better segregation ability. Nevertheless, the post-chemotherapy group showed contradictory results. One index showed decreased values, and two indices showed increased values.

The measurement of segregation quantified the presence of clusters or modules. In fact, two versions of the clustering coefficient exist: local and global. In the present study, the clustering coefficient indicated the local clustering coefficient, which calculated the clustering of a single node. For transitivity, a classical variant of the clustering coefficient, the calculation was based on triplets of nodes and gave an indication of global clustering in the whole network. Both indices can be expressed as probabilities. The difference is how they sample the random nodes. Estrada and Schank et al. provide evidence that the clustering coefficient and transitivity can produce widely different results in some circumstances (62–64). Modularity is a more sophisticated measure of network segregation. It measures the strength of the division of a network into modules and reflects the concentration of edges within modules. Higher values represent dense interconnections between the nodes within modules but sparse interconnections in different modules (33, 62).

It has been proposed that brain structure facilitates and constrains functional interactions within brain networks. White matter integrity facilitates local interconnectivity and interferes with distributed connectivity. With disrupted white matter integrity, more information might be lost during transmission. This causes neurons to recruit more resources from nonlocal areas. That is, it relies more on distributed neural processing. From a network perspective, a stable structural network supports dynamic functional integration to address various task demands (21, 65, 66). In line with our study, Bruno et al. also reported a lower clustering coefficient in breast cancer patients and suggested that breast cancer and postchemotherapy issues may result in disruption in the topological organization of the brain network (67).

Integrated the results of our study, including decreased white matter integrity, weaker subnetwork interconnections, and reduced local clustering coefficient and modularity in the post-chemotherapy group. We suggest that chemotherapeutic regimens, via widespread destruction of small-range white matter integrity, may affect overall network stability and lead to alterations in brain function.

### Correlation analysis

In this longitudinal study, we detected several relationships between changes in GFA/NQA values and changes in NP test/PRO scores. For mood symptoms, higher PHQ9 and HADS-A scores represent higher levels of depression and anxiety, respectively. We found significant negative correlations between PHQ9 and the regions of the PLIC, SFG, MFG and CC. In addition, HADS-A was negatively correlated with SFG. These regions were found to be associated with emotions.

Both the CC and frontal lobe play a key role in regulating emotional processing and multiple cognitive functions. Studies have investigated patients with impairment of white matter integrity in the frontal gyrus and CC who may lose various brain functions and suffer from mood disorders (40, 68). In addition to depression, correlations between poorer cognition and disrupted white matter integrity were detected in this study. Abraham et al. reported a positive correlation between FA in the CC and processing speed assessed by the DSS (39). Deprez et al. explored frontal gyrus integrity negatively associated with the Cognitive Failures Questionnaire (CFQ) (40). The white matter integrity in the CC as well as the MFG and anxiety level were reduced from pre- to post-chemotherapy. The correlation between these regions and mood symptoms remained negative. It has been suggested that chemotherapy does not destroy these relationships, and some patients experience not only chemotherapeutic neurotoxicity but also white matter recovery or posttraumatic growth (PTG) (15, 69, 70). The corticospinal tract (CST) constitutes a large part of the internal capsule and plays an important role during nerve conduction ascending and descending. The literature indicates that CST could be an indicator of major depressive disorder (MDD) patients who have symptoms of emotional disturbance and cognitive impairment (71, 72). Furthermore, Karahan et al. revealed a link between white matter microstructure and sensorimotor speed (73). Our results showed that a greater reduction in white matter microstructure was correlated with gloomier and poorer cognitive performance. Damage to the CST was also mentioned in breast cancer patients with chemotherapy (14).

For cognitive ability, the results were highly repetitive in both objective and subjective cognitive assessments. We observed that a greater reduction in cognition was associated with more white matter integrity in the regions of the CC, SLF, PCG, PLIC, and putamen. The CC and PLIC were discussed above. The SLF contributes to a broad range of cognitive abilities, such as visuospatial nonverbal cognition and verbal memory. Rehabilitation of cognitive function in patients with neurological disorders may be impacted by these relationships. Furthermore, white matter integrity changes in the SLF have previously been linked to chemotherapy (14, 17, 74, 75). The putamen is critical in episodic memory, motor control and learning. Recent studies have suggested that the putamen is also involved in attentional processes. In addition, the putamen is part of the striatum, and the pathological state within the striatum can manifest clinical symptoms ranging from motor dysfunction to various psychiatric disorders (76, 77). Dumas et al. reported that functional connectivity between the putamen and DAN is decreased a month after chemotherapy but recovers to baseline after a year (50). In the present study, the wide range of days after receiving chemotherapy in the post-chemotherapy group might have canceled out the effects of neurotoxicity and recovery on white matter integrity. Therefore, we could not identify significant changes in the paired t test. However, relationships between reductions in GQI indices in the putamen and declines in cognitive performance in attention and processing speed associated with chemotherapy were evident in the correlation analysis. There was a correlation between mild cognitive impairment and disruption of the PCG, one of the functional hubs in the DMN (78). Tong et al. reported longitudinal decreases in FA as well as metabolism in the PCG assessed by DTI and MRS. These decreases were correlated with memory decline (42). In the present study, we did not see a significant difference in PCG in the longitudinal design. Nonetheless, the relationships between longitudinal changes in PCG and cognitive performance were evident. We also observed significant decreases in subjective cognitive function.

### Limitations

The strengths of this study were combined analyses in white matter microstructure and macroscale with longitudinal design. Moreover, we examined the changes between GQI indices and NP tests/PR. However, there are a few limitations to this study. First, the high attrition rate in the follow-up assessment led to a small sample size in the longitudinal study. Second, we did not differentiate subtypes of breast cancer, chemotherapeutic regimens, other cancer treatment strategies, or menopausal status. Third, there was a mixture of acute, late, and recovery effects of chemotherapy due to the wide range of days following completion of chemotherapy. Last, the neuropsychological tests did not comprehensively evaluate more aspects of cognition. Furthermore, the incomplete NP test/PR in HCF resulted in a lost controlled experiment. Future studies could recruit a larger sample size and distinguish the most harmful drugs in neurotoxicity. Additionally, mediation analysis is needed to clarify the causal relationship.

## Conclusion

Injuries to myelin, whether caused by a traumatic stressor or chemotoxicity, can adversely affect cognition. The present study investigated cerebral white matter microstructure and macroscale alterations in breast cancer survivors with and without chemotherapy. Our results suggested that the patients had changes in local white matter integrity and network performance in the frontal lobe connection after chemotherapy. Additionally, the longitudinal changes in white matter integrity were correlated with changes in neuropsychological tests and questionnaires. These results provided evidence of white matter alterations in breast cancer patients, which may serve as potential imaging markers of cognitive changes. In the future, the study may be beneficial to create and evaluate strategies designed to maintain or improve cognitive function in breast cancer patients undergoing chemotherapy. These strategies could encompass cognitive training programs, lifestyle adjustments, or pharmacological methods, with the potential to alleviate or reverse cognitive impairment associated with chemotherapy over time.

## Data availability statement

The raw data supporting the conclusions of this article will be made available by the authors, without undue reservation.

## Ethics statement

The studies involving humans were approved by the Institutional Review Board of Chang Gung Memorial Hospital, Chiayi, Taiwan (No. 104-5082B, 201700256B0, 201702027B0). The studies were conducted in accordance with the local legislation and institutional requirements. The participants provided their written informed consent to participate in this study.

## Author contributions

VC: Conceptualization, Data curation, Funding acquisition, Resources, Writing – review & editing. WC: Formal analysis, Investigation, Software, Visualization, Writing – original draft. Y-HT: Data curation, Resources, Writing – review & editing. RM: Writing – review & editing. J-CW: Conceptualization, Funding acquisition, Methodology, Project administration, Supervision, Validation, Writing – review & editing, Writing – original draft.
