# Supplementary material for: Longitudinal assessment of chemotherapy-induced brain connectivity changes in cerebral white matter and its correlation with cognitive functioning using the GQI
Source: Front Neurol. 2024 Feb 7;15:1332984. doi: 10.3389/fneur.2024.1332984 (PMC10879440; doi:10.3389/fneur.2024.1332984)
Supplement: Supplementary file 1 [file Data_Sheet_1.docx]

**Table S3. Appendix**

CICI: chemotherapy-related cognitive impairment

CRCI: cancer-related cognitive impairment

GQI: generalized q-sampling imaging

BB: pre-chemotherapy group

HC: noncancer control group

BBF: follow up of BB group after chemotherapy; post-chemotherapy groups

HCF: follow up of HC group

GTA: graph theoretical analysis

DMN: default mode network

CC: corpus callosum

MFG: middle frontal gyrus

PLIC: posterior limb of the internal capsule

SLF: superior longitudinal fasciculus

WHO: World Health Organization

BNC: breast cancer

CNS: central nervous system

MRI: magnetic resonance imaging

SDF: spin distribution function

GFA: generalized fractional anisotropy

NQA: normalized quantitative anisotropy

DAN: dorsal attention network

TP1: time point one

TP2: time point two

NP tests: neuropsychological tests

CTT: Color Trails Test

DSS: Digit Symbol Substitution

PR: patient-reported cognitive function and mood symptoms

FACT-cog: functional assessment of cancer therapy cognitive

CogPCI: cognitive impairment

CogPCA: cognitive ability

CogOth: cognitive impairment by others

CogQoL: impact on quality of life

PHQ-9: Patient Health Questionnaire-9

DSM-IV: Diagnostic and Statistical Manual of Mental Disorders

HADS-A: Anxiety subscale of the Hospital Anxiety and Depression Scale

SE-EPI: spin echo echo-planar imaging

TR: repetition time

TE: echo time

FOV: field of view

NEX: number of excitations

DEI: diffusion-weighted imaging

SPM statistical parametric mapping

MNI: Montreal Neurological Institute

VBA: voxel-based analysis

FDR: false discovery rate

AAL: automated anatomical labeling

NBS: network-based statistics

AUC: area under the curve

GLM: general linear model

ORBmidF: orbital part of the middle frontal gyrus

IFG: left inferior frontal gyrus

STG: superior temporal gyrus

MTG: middle temporal gyrus

SFG: superior frontal gyrus

PCG: posterior cingulate gyrus

VBM: voxel-based morphometry

DTI: diffusion tensor imaging

FA: fractional anisotropy

MD: mean diffusivity

RD: radial diffusivity

fMRI: functional MRI

rs-fMRI: resting-state fMRI

SCA: seed-based correlation analysis

CEN: central executive network
CFQ: Cognitive Failures Questionnaire

PTG: posttraumatic growth

CST: corticospinal tract

MDD: major depressive disorder
