# Supplementary material for: Longitudinal assessment of chemotherapy-induced brain connectivity changes in cerebral white matter and its correlation with cognitive functioning using the GQI
Source: Front Neurol. 2024 Feb 7;15:1332984. doi: 10.3389/fneur.2024.1332984 (PMC10879440; doi:10.3389/fneur.2024.1332984)
Supplement: Supplementary file 2 [file Data_Sheet_1.pdf]

Table S1. Summary of repeated-measures ANCOVA results

Region	MNI coordinates (x, y, z)	Peak F or T scores	Effect size	Figure
group x time effect		F scores		
corpus callosum (CC)	6, 20, 10	14.6	0.76	Fig. 1-a, b
bilateral middle frontal gyrus, orbital (ORBmidF)	±18, 46, -8	14.3	0.71	Fig. 1-c, d
bilateral middle frontal gyrus (MFG)	±44, 8 ,34	14.4	0.73	Fig. 1-e
paired t-test (BB>BBF)		T scores		
corpus callosum (CC)	8, 22, 0	4.2	0.73	Fig. 2-a, c
	-16, 28, 18	4.7	0.78	Fig. 2-b, d
left middle frontal gyrus, orbital (ORBmidF)	-18, 44, -10	4.1	0.71	Fig. 2-e, f
left middle frontal gyrus (MFG)	-32, 12, 36	4.8	0.80	Fig. 2-g
left inferior frontal gyrus (IFG)	-36, 24, 14	4.6	0.77	Fig. 2-h
right superior temporal gyrus (STG)	38, -24, 6	4.6	0.76	Fig. 2-i
left middle temporal gyrus (MTG)	-52, -6, 20	4.4	0.75	Fig. 2-j
bilateral insula	±42, 2, -2	4.3	0.74	Fig. 2-k
paired t-test (HC>HCF)		T scores		
right inferior frontal gyrus (IFG)	38, 0, 26	4.3	0.73	Fig. 3-a
left superior temporal gyrus (STG)	-44, -22, -4	4.8	0.79	Fig. 3-b

Results in voxel-based analysis were presented in coordinate of Montreal Neurological Institute (MNI) space.

Table S2. Summary of correlation analysis results

Region	MNI coordinates (x, y, z)	Correlation coefficient	Figure
PHQ9			
left posterior limb of internal capsule (PLIC)	-22, -8, 6	-0.468	Fig. 6-a, b
right superior frontal gyrus (SFG)	24, 20, 64	-0.600	Fig. 6-c, d
left middle frontal gyrus (MFG)	-44, 22, 42	-0.449	Fig. 6-e
corpus callosum (CC)	-16, 20, 26	-0.337	Fig. 6-f
HADS-A			
right superior frontal gyrus (SFG)	12, 54, 8	-0.566	Fig. 6-g, h
CTT1			
bilateral posterior limb of internal capsule (PLIC)	±16, -8, 12	-0.395	Fig. 7-a
left posterior limb of internal capsule (PLIC)	-16, -12, 10	-0.506	Fig. 7-b
left superior longitudinal fasciculus (SLF)	-30, 12, 20	-0.541	Fig. 7-c
left putamen	-26, 10, 2	-0.563	Fig. 7-d, e
CTT2			
right posterior cingulate gyrus (PCG)	10, -48, 2	-0.433	Fig. 7-f
right posterior limb of internal capsule (PLIC)	22, -24, 18	-0.438	Fig. 7-g
DSS			
right posterior limb of internal capsule (PLIC)	14, -4, 10 / 18, -14, 12	0.352	Fig. 7-h, i
corpus callosum (CC)	14, 20, 18	0.332	Fig. 7-j
right putamen	26, 10, 0	0.461	Fig. 7-k, l
FACT-cog			
left posterior cingulate gyrus (PCG)	-16, -54, 22	0.658	Fig. 8-a, b
left posterior limb of internal capsule (PLIC)	-26, -18, 16	0.586	Fig. 8-c
CogPCI			
left middle frontal gyrus (MFG)	-32, 24, 48	0.246	Fig. 8-d
left posterior cingulate gyrus (PCG)	-16, -54, 22	0.578	Fig. 8-e, f
bilateral superior longitudinal fasciculus (SLF)	±34, -18, 30	0.528	Fig. 8-g, h
CogPCA			
left posterior limb of internal capsule (PLIC)	-16, -6, 6	0.321	Fig. 8-i

Results in voxel-based correlation analysis were presented in coordinate of Montreal Neurological Institute (MNI) space. Please look for the abbreviation details in the appendix.
